# Topical zoledronic acid decreases micromotion induced bone resorption in a sheep arthroplasty model

**DOI:** 10.1186/s12891-017-1802-z

**Published:** 2017-11-13

**Authors:** Thomas Jakobsen, Søren Kold, Juan Shiguetomi-Medina, Jorgen Baas, Kjeld Soballe, Ole Rahbek

**Affiliations:** 1 Orthopaedic Research Laboratory, Department of Orthopaedics, Aarhus University Hospital, Norrebrogade 44, Building 1A, DK-8000 Aarhus, Denmark; 2 Department of Orthopaedics, Aalborg University Hospital, Aalborg, Denmark

**Keywords:** Bisphosphonate, Osseointegration, Implant, Animal, Arthroplasty

## Abstract

**Background:**

Initial micromotion of a total hip replacement is associated with aseptic loosening. The use of bisphosphonates could be one way to reduce peri-implant bone resorption induced by micromotion. Bisphosphonates compounds are inhibitors of bone resorption. The aim of this study was to investigate whether local treatment with bisphosphonate would reduce bone resorption and fibrous tissue around an experimental implant subjected to micromotion.

**Methods:**

One micromotion implant were inserted into each medial femoral condyle in ten sheep. During each gait cycle the implant axially piston 0.5 mm. During surgery one of the femoral condyles were locally treated with 0.8 mg zoledronate. The other condyle served as control. Observation period was 12 weeks.

**Results:**

Histological evaluation showed a fibrous capsule around both the control and bisphosphonate implants. Histomorphometrical analysis showed that 97% of the surface on both control and bisphosphonate implants were covered by fibrous tissue. However, the bisphosphonate was able to preserve bone in a 1 mm zone around the implants.

**Conclusion:**

This study indicates that local treatment with bisphosphonate cannot prevent the formation of a fibrous capsule around an implant subjected to micromotion, but bisphosphonate is able to reduce resorption of peri-prosthetic bone.

## Background

Initial stable implant fixation is important for long-term survival of total joint replacements [1, 2]. Radiostereometrical studies indicate that early migration is a associated with later revision [1–3]. Treatment strategies that optimize initial implant fixation and prevents early implant migration could have the potential to reduce risks of later revision.

Failure of initial implant osseointegration will result in early implant migration and bone resorption [4]. Early implant migration is associated with reduced resistance to withstand implant movement induced by external load [5]. Continues implant migration is associated with implant failure [3]. Early bone resorption can be initiated by an unstable implant generating micro-movement or fluid pressure [6–8]. We have previously shown that implant micromotion without the presence of wear debris can generate a fibrous membrane and cause bone resorption [9]. In vivo studies suggest that instability induced bone resorption works through similar molecular mechanisms as particle induced bone resorption [10, 11]. Aseptic loosening of an implant involves several steps. One of the first steps could be bone resorption due to micro-movement of the implant. The resorption bone is replaced with fibrous tissue. Wear debris will subsequently migrate from the joint articulation along the fibrous tissue to the bone to implant interface and aggravate the process [12, 13]. Longevity of the implant might potentially be increased if micro-movement of the implant were not allowed to induce bone resorption.

One way to reduce micromotion induced bone resorption and fibrous membrane formation could be with the use of bisphosphonates. These compounds binds strongly to bone, inhibit osteoclastic activity, and reduce bone resorption [14]. Systemic bisphosphonate treatment has previously in an experimental model of osteolysis been able to reduce bone resorption [15]. We have previously shown that local treatment with bisphosphonate can increase new bone formation, preserve lamellar bone and allograft, and increase biomechanical implant fixation in an experimental joint replacement model [9]. In a clinical study, local treatment with bisphosphonate was shown to reduce acetabular component migration measured by radiostereometrical analysis [16]. The same effect has been observed in the study investigating local ibandronate treatment in total knee arthroplasties [17]. Others have shown the local or systemic treatment with bisphosphonates can reduce but not prevent osteolytic bone resorption and formation of fibrous tissue in a rat model of prosthetic loosening [18, 19]. Our group has previously shown in a large animal model that systemic alendronate can reduce bone resorption around unstable implants but not prevent it [20]. A stronger stimulus might be needed. Only way to obtain a stronger stimulus could be with topical administration of a third generation bisphosphonate. The aim of this study was to investigate in a sheep arthroplasty model whether topical treatment with bisphosphonate would reduce bone resorption and fibrous tissue.

We tested the hypothesis that local zoledronate treatment would reduce bone resorption and fibrous tissue formation around implants subjected to micromotion.

## Methods

### Study design

We used 10 skeletally mature Danish Landrace sheep with a mean weight of 40 kg (range, 35–50 kg). The sheep were obtained though our Institutional farm (Påskehøjgaardcentret, 8380 Trige, Denmark) affiliated with our Research facility. Our Institutional Animal Care and Use Committee approved the study. Institutional guidelines for the treatment and care of experimental animals were followed.

**Fig. 1:**
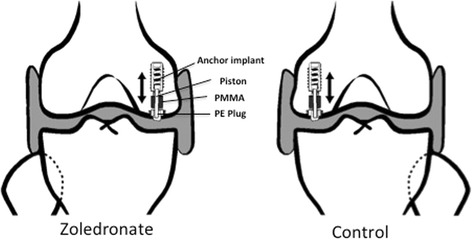
Schematic drawing showing implant position in the medial femoral condyle. Reproduced with permission from copyright holder/author

In this study, we used a paired design with intervention and control group in the same sheep. We have previously shown that the used model of micromotion is able to induce bone resorption [9]. In each sheep, we inserted one loaded micromotion device (Fig. 1) into each of the medial femoral condyles. Zoledronate was administered locally in one of the knees. Saline was used as control in the contralateral knee. The zoledronate and control implants were systematically alternated between left and right knee. Our observation period was 12 weeks.

**Fig. 2:**
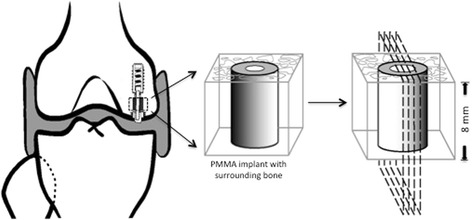
Schematic diagram showing the specimen preparation. Each bone-implant specimen is embedded and cut into four slides for histomorphometrical analysis. Reproduced with permission from copyright holder/author

**Fig. 3:**
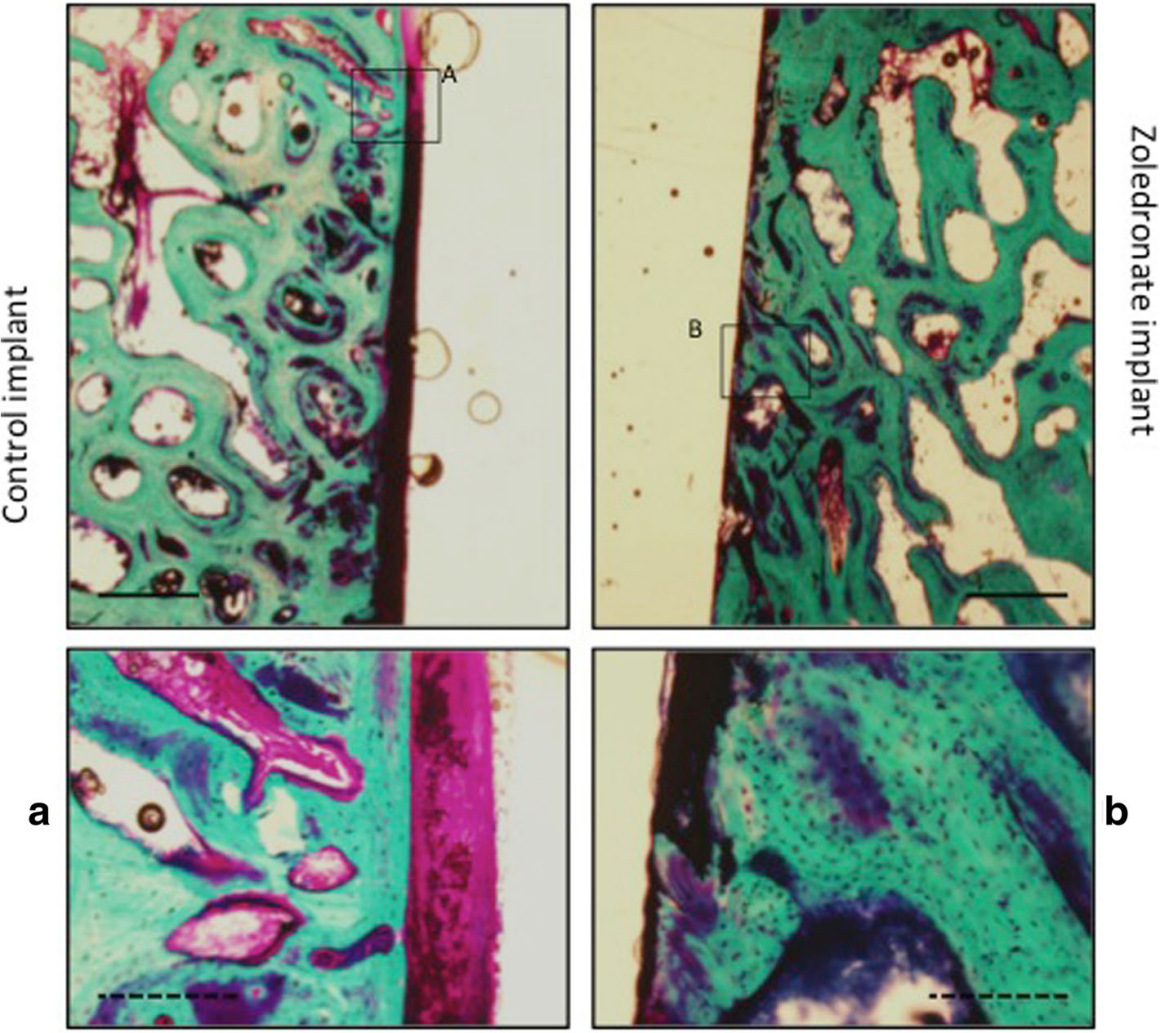
Representative photomicrographs of samples from the same animal. The samples were stained with basic fuchsin and counterstained with 2% light green. Note the thick fibrous membrane around the control implant compared to the zoledronate implant. Solid bar = 1 mm. Dotted bar = 0.3 mm

**Fig. 4:**
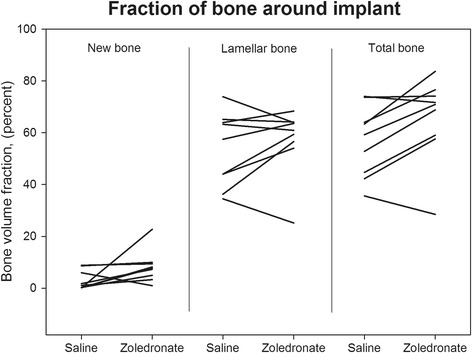
Tissue-volume fractions in a 0–1 mm zone around implants. Paired data connected by line

Our sample size calculation was based on a previous study [9]. We assumed the standard deviation of the relative change to be 50%. Two-sided α and β were set to 5% and 20%, respectively. We added two extra animals to the calculated sample size of eight to counteract if implants from one or two animals were lost for subsequent analysis.

### Implants

We used 20 custom-made micromotion implant devices (Fig. 1). Our micromotion device has previously been described in detail [9, 21]. In short, the micromotion device allows a polymethylmethacrylate (PMMA) implant to piston 0.5 mm with respect to the bone during each step the animal takes. The micromotion creates shear forces in the bone to implant interface. The implants in this study were made sterile by gamma irradiation (25–50 kGy for 16 h, Codan Steritex, Espergaerde, Denmark).

### Surgery

With the sheep under general anesthesia, we used our standard anteromedial surgical approach to the knee [9, 21, 22]. Our micromotion device was inserted into the weight-bearing portion of the medial femoral condyle. The procedure has been described in detail in our previously study [9]. During each surgery, we tested and assured that the micromotion device would piston during each gait cycle.

Before the PMMA implant was mounted onto the anchor screw, we soaked the bone cavity for 60 s with either saline as control or 1 mL saline containing 0.8 mg zoledronate (Aclasta, Novartis Healthcare A/S, Copenhagen, Denmark) as intervention. After soaking the bone for 60 s, the cavity was rinsed with saline and excess zoledronate or saline together with blood coming from the marrow cavity was sucked away. In this study, all sheep were weight bearing on the operated leg within 5 days of surgery. After the 12 weeks observation period the sheep were euthanized with an overdose of hypersaturated barbiturate and the bones were collected for preparation and analyses. At harvest, all pistons were movable.

### Specimen preparation

Bone specimens were stored at −20 °C immediately. We cut each specimen perpendicular to the long axis of the implant using a water-cooled bad saw (Exact Apparatebau, Nordenstedt, Germany) (Fig. 2). The specimens was fixed in 70% ethanol and used for later analysis. Preparation and subsequent evaluation were blinded.

### Histology and Histomorphometry

All bone-implant specimen were dehydrated in ethanol (70%–96%) containing basic fuchsin, and embedded in Epoxy (EPOFIX, Struers, Copenhagen, Denmark). Four 25-μm thick sections were cut with a hard tissue microtome (KDG-95, MeProTech, Heerhugowaard, The Netherlands) around the center part of each implant [23]. The sections were cut with a distance of 400 μm. Before making the sections, the implant was randomly rotated around its long axis. The sections were cut parallel to this axis. These techniques provide reliable results with negligible bias [24]. Before the sections were mounted on glass, we surface stained them with 2% light-green (BDH Laboratory Supplies, Poole, England).

We used a stereological software program (newCAST, Visiopharm A/S, Horsholm, Denmark) for the histomorphometrical analysis. Volume fractions of bone were estimated by point counting [25] in a zone from the implant surface and 1 mm into the surrounding bone. Fractions of bone in contact with the implant surface were estimated using sine-weighted lines [26].

### Statistical analysis

Intercooled Stata 9.0 (Stata Inc., College Station, TX, USA) were used for statistical analysis. Statistical analyses were done on ratios between paired data, which were not normally distributed. All variables were log-transformed and Student’s paired t-test was performed on absolute differences between normally distributed log-transformed paired data. An absolute difference between the logarithms of a pair of data equals the logarithm of the ratio within the pair [27].

## Results

### Surgery

One sheep died during surgery and was excluded. The remaining 9 sheep completed the 12-weeks observation period. No clinical signs of infection were present at time of euthanization.

### Histology

The most striking difference between the control and zoledronate implants was the presence of a 200–300 μm thick fibrous membrane around the control implants (Fig. 3). A fibrous membrane was also present around the zoledronate implants, but the membrane was thinner with an approximately thickness of 50 μm. The fibrous membrane in both the control and zoledronate group consisted of dense paralleled fibers. No qualitative morphological difference was found between the two groups when looking at the bone in the peri-implant zone. The bone consisted mainly of lamellae with spindle-shaped osteocytes in between. Sparse amount of osteoclasts were observed in both groups. No retained bone debris was observed in any of the groups.

### Histomorphometrical analysis

The local zoledronate treatment preserved 14% (*p* = 0.02) more total bone around the implants in a 1 mm zone in the zoledronate group compared to the control group (Fig. 4). We found a total peri-implant bone volume fraction of 57% (95% CI: 46% - 67%) in the control group and 66% (95% CI: 53% - 78%) in the zoledronate group. No statistically significant changes were found when comparing bone volume fractions for woven (*p* = 0.08) or lamellar (*p* = 0.32) bone (Fig. 3). In the zoledronate group, 16% (95% CI: 2% - 29%) of 1 mm peri-implant zone was made of fibrous tissue compared to 23% (95% CI: 14% - 33%) in the control group (*p* = 0.22).

The implant surfaces in the both the zoledronate and control group were virtually covered by fibrous tissue. The surface fraction for fibrous tissue was 97% (95% CI: 94%- 100%) in the zoledronate group compared to 97% (95% CI: 94% - 99%) in the control group (*p* = 0.65). We found no significant differences in the surface-fractions of woven, lamellar, and total bone (*p* = 0.76, *p* = 0.42, *p* = 0.71).

## Discussion

The purpose of this study was to investigate whether topical treatment with zoledronate could prevent bone resorption and fibrous tissue formation in our large animal model of micromotion induced bone resorption. We found that zoledronate did not prevent formation of a fibrous membrane, but was able to reduce bone resorption and thickness of the fibrous membrane.

The used model is intended to imitate the bone to cement interface of a total hip replacement. We have previously demonstrated that our experimental model are able to induce peri-implant bone resorption and therefore also suitable to test local adjuvant therapies against bone resorption [9]. The paired design allowed us to eliminate the biological difference between individuals.

Our study is limited by an observation period of 12 weeks and only nine animals completed the study. Non-significant differences should therefore be interpreted with caution.

Zoledronate is a potent inhibitor of bone resorption [28]. We have previously shown that local treatment with zoledronate can inhibit resorption of allograft and increase fixation of both primary and revision implants [29, 30]. In this study, we show that local treatment with zoledronate can reduce resorption of bone in a 1 mm peri-implant zone around an implant subjected to controlled micromotion.

Our model of micromotion has a piston that allows a 0.5 mm movement of the implant during each loading. The model was designed to imitate a cemented femoral implant subjected to micromotion. The amplitude of movement of our implant is above the accepted threshold of 0.15 mm studies and suggested by Van der Voort based on RSA and thereby comparable to clinical implants with a high risk of as aseptic loosening [31]. Furthermore, the loading conditions of both clinical femoral implants and our experimental implants are comparable; both implants transfers load by shear forces though the bone-cement imterface. In our model, movement will occur at the implant-to-bone interface during each gait cycle and thereby create high shear forces and strain between the implant and tissue in contact with the implant. According to studies by and Carter and Giori, the amplitude of strain dictates which tissue can be formed [32, 33]. A high strain will induce bone resorption and formation of fibrous tissue. We found that local zoledronate treatment were able to partly counteract the strain/micromotion induced bone resorption and preserve 14% more total bone compared to our control.

An interesting histological finding is the difference in thickness of the fibrous membrane between the zoledronate and control groups. We know from previous studies from our group that micromotion is able to induce formation of a fibrous membrane [9, 34]. In this study we observed that local zoledronate treatment histologically reduced the thickness of the membrane. It could be that shear forces and stain at the tissue-to-implant interface are to high to by counteracted by zoledronate. Further away form the implant surface, strain and shear forces diminishes and zoledronate are able to partly preserve bone. We know from previous studies that increased amount of bone and decreased amount of fibrous tissue in correlated to increased implant stability [22]. The difference in fibrous layer thickness should therefore have the potential to make the zoledronate implants more mechanically stable.

It has previously been shown that systemic alendronate is able to inhibit bone resorption in a canine osteolytic hip arthroplasty model [15]. An animal study using a rodent model has shown that systemic treatment with alendronate or clodronate can reduce instability-induced bone resorption, but high doses are needed [19]. One likely explanation for the preserved bone volume density found in this study is the inhibitory effect of zoledronate on bone resorption. Previous studies have shown that preservation of lamellar bone often leads to increased formation of new bone [35–37]. The increased new bone formation is explained by increased osteoconductive properties of the preserved lamellar bone. In this study, we are not able to show a statistically significant increase in peri-implant formation of new bone (8% in zoledronate group vs. 3% in control group, *p* = 0.08). One possible explanation could be a to short observation period for new bone formation to occur. The effect on bone formation and resorption has been shown to be different over time [37]. A longer observation period might have shown a more pronounced effect of zoledronate on both bone formation and resorption.

Local treatment with zoledronate was not able to prevent formation of peri-implant fibrous tissue. However, zoledronate was able to reduce the thickness of the peri-implant fibrous membrane. This is in agreement with results from another study where local alendronate treatment reduced formation of soft tissue, but did not prevent its formation [19]. It is also in agreement with a clinical study were local ibandronate treatment reduced occurrences of radiolucent lines around acetabular cups lines, but not prevented them [16]. Based on our previous study were systemic alendronate reduced bone resorption around an unstable implant, we expected that a stronger anti-resorptive stimulus could be obtained with local zoledronate. We therefore expected that local zoledronate could prevent bone resorption and formation of fibrous tissue. It may be that implant micromotion is a too strong stimulus for even local zoledronate being able to completely prevent bone resorption. Another explanation could be that the single dose of zoledronate acts only as a defense again bone-resorption for a limited amount of time. Zoledronate, released from bone by osteoclastic resorption, could be slowly washed away from the implant-bone interface. By time the concentration will be too low to inhibit the continuous strong bone resorptive stimulus from micromotion.

In clinical practice, soaking the bone bed before implantation is a simple procedure. We know from a previous studies that the effect on implant osseointegration of local bisphosphonate is dose-dependent [29, 38]. Bisphosphonate bound to bone will only exert its effect on the osteoclast during bone resorption and intra-cellular internalization [39]. However, any cell, including bone-forming cells, can internalize unbound bisphosphonate. Removal of unbound bisphosphonate by irrigating the bone bed after soaking it with bisphosphonate is therefore of importance.

## Conclusion

In conclusion, this study indicates that local treatment with zoledronate can reduce micromotion induced bone resorption, but not prevent it. Studies investigating the effect of different zoledronate doses and observation periods are needed.

## Abbreviations

PE: polyethylene
PMMA: polymethylmethacrylate
RSA: Radiostereometric Analysis
